# Association between mortality and critical events within 48 hours of transfer to the pediatric intensive care unit

**DOI:** 10.3389/fped.2023.1284672

**Published:** 2023-12-21

**Authors:** Huan Liang, Kyle A. Carey, Priti Jani, Emily R. Gilbert, Majid Afshar, L. Nelson Sanchez-Pinto, Matthew M. Churpek, Anoop Mayampurath

**Affiliations:** ^1^ Department of Biostatistics & Medical Informatics, University of Wisconsin-Madison, Madison, WI, United States; ^2^ Department of Medicine, University of Chicago, Chicago, IL, United States; ^3^ Department of Pediatrics, University of Chicago, Chicago, IL, United States; ^4^ Department of Medicine, Loyola University Medical Center, Maywood, IL, United States; ^5^ Department of Medicine, University of Wisconsin-Madison, Madison, WI, United States; ^6^ Department of Pediatrics (Critical Care), Ann & Robert H. Lurie Children’s Hospital of Chicago, Chicago, IL, United States

**Keywords:** pediatrics, intensive care unit, critical events, mortality, electronic health records

## Abstract

**Introduction:**

Critical deterioration in hospitalized children, defined as ward to pediatric intensive care unit (PICU) transfer followed by mechanical ventilation (MV) or vasoactive infusion (VI) within 12 h, has been used as a primary metric to evaluate the effectiveness of clinical interventions or quality improvement initiatives. We explore the association between critical events (CEs), i.e., MV or VI events, within the first 48 h of PICU transfer from the ward or emergency department (ED) and in-hospital mortality.

**Methods:**

We conducted a retrospective study of a cohort of PICU transfers from the ward or the ED at two tertiary-care academic hospitals. We determined the association between mortality and occurrence of CEs within 48 h of PICU transfer after adjusting for age, gender, hospital, and prior comorbidities.

**Results:**

Experiencing a CE within 48 h of PICU transfer was associated with an increased risk of mortality [OR 12.40 (95% CI: 8.12–19.23, *P* < 0.05)]. The increased risk of mortality was highest in the first 12 h [OR 11.32 (95% CI: 7.51–17.15, *P* < 0.05)] but persisted in the 12–48 h time interval [OR 2.84 (95% CI: 1.40–5.22, *P* < 0.05)]. Varying levels of risk were observed when considering ED or ward transfers only, when considering different age groups, and when considering individual 12-h time intervals.

**Discussion:**

We demonstrate that occurrence of a CE within 48 h of PICU transfer was associated with mortality after adjusting for confounders. Studies focusing on the impact of quality improvement efforts may benefit from using CEs within 48 h of PICU transfer as an additional evaluation metric, provided these events could have been influenced by the initiative.

## 1. Introduction

In pediatric hospitals, determining the impact of clinical interventions is challenging as the rates of adverse events such as mortality and cardiac arrests are lower in children than adults (1–6). Approximately ten years ago, Bonafide et al. proposed a metric called “critical deterioration”, defined as a ward-to-intensive care unit (ICU) transfer followed by non-invasive or invasive mechanical ventilation (MV) or vasoactive infusion (VI) within 12 h (6). Critical deterioration (CD) events were associated with increased mortality risk through univariable analysis and also offered sufficient statistical power to analyze the impact of implementation studies. In a follow-up study, Bonafide et al. demonstrated the effectiveness of pediatric rapid response systems tasked with early recognition and response to deterioration in reducing CD events (7).

Since its inception, the CD metric has been incorporated by several pediatric outcomes research studies. For example, studies have reported incidences of CD in patient sub-populations (8), profiled patients experiencing CD (9), determined the impact of CD (10, 11), and used CD events to measure implementation of care improvement strategies (12, 13). CD events have also been used as outcomes for evaluating the performance and effectiveness of pediatric early warning scores and risk prediction models (14–16).

Although the use of CD events as an evaluation metric has become routine, the rates of CD events are still low, particularly in non-quaternary care pediatric hospitals. Therefore, studies evaluating clinical interventions, such as assessing the impact of ward or emergency-based clinical decision support tools in tertiary care settings, might benefit from analyzing alternative metrics. For example, while the CD event definition is limited to the first 12 h of PICU transfer, there is evidence in the scientific literature that events comprising the definition, i.e., MV or VI events, can occur any time after transfer (17). Additionally, CD events pertain to transfers from the ward, as the intended goal was to improve the early recognition of deterioration among hospitalized children. However, recent studies have implemented early warning systems and clinical decision support tools within the pediatric emergency department (ED), focusing on further improving pediatric outcomes (18–20). Therefore, ward- and ED-based implementation studies could benefit from the additional statistical power of using an evaluation metric centered on MV or VI events that happen in the PICU 12 h after transfer. However, the association between events that occur beyond the first 12 h of PICU transfer and in-hospital mortality is not well understood.

The objective of this study was to determine the association between in-hospital mortality and experiencing critical events (CEs, i.e., an MV or VI event) within 48 h of being transferred to the PICU from the ward or ED after adjusting for potential confounders. We further explore the association at different time points from PICU transfer and across patient sub-populations and event types. We hypothesize that CEs within the first 48 h elevate the risk of in-hospital mortality, thereby providing an additional metric to evaluate the effectiveness of hospital-wide quality improvement studies.

## 2. Materials and methods

### 2.1. Setting and study population

We conducted a retrospective observational cohort study of all pediatric patients, i.e., age <18 years, who transferred directly to the PICU from the ED or ward at two tertiary care centers: the University of Chicago Comer Children's Hospital and Loyola Medicine Ronald McDonald Children's Hospital from 2009 to 2019. Patients who experienced either invasive mechanical ventilation or vasoactive infusion prior to PICU transfer were excluded from the analysis. Data were collected from the electronic health record (EHR; Epic, Verona, WI) and hospital administration databases. The study was approved by local Institutional Review Boards (University of Chicago IRB# 18-0645; Loyola University IRB# 215464).

### 2.2. Outcome and exposure variables

Our primary outcome was in-hospital mortality. Our primary exposure was the occurrence of the first CE within 48 h of being transferred to the PICU. CEs beyond the first 48 h of PICU transfer were not considered, as these are more likely from the progression of the underlying disease or a new illness when under the care of the PICU team and thus may not be a valid target for quality initiatives focused on pre-transfer care (21, 22). Initiation of invasive mechanical ventilation was identified based on a new flowsheet recording of invasive mechanical ventilation for a patient previously on non-invasive ventilation (e.g., room air, nasal cannula, etc.). Only invasive ventilation was considered to align with more recent definitions of pediatric deterioration (23). We considered vasoactive infusion to include standard vasopressors and inotropes (dobutamine, dopamine, epinephrine, milrinone, norepinephrine, and vasopressin) which were identified from the medication administration data.

### 2.3. Statistical analysis

Patient characteristics and outcome differences between children who experienced at least one CE after PICU transfer and patients who did not were analyzed using appropriate *t*-tests or Wilcoxon's non-parametric test for numeric variables and chi-squared tests for categorical variables. We then conducted a cumulative frequency analysis on CEs to examine their occurrence distribution throughout the PICU stay. Briefly, we divided data from the point of PICU stay for a patient into 12-h intervals until 48 h. These time intervals were chosen based on the original definition of CD, which focused on a 12-h interval and the frequency of CEs being large enough to conduct meaningful analysis. Next, we calculated the cumulative proportion of patients experiencing their first CE for each time interval. Finally, cumulative frequency analysis was repeated using initial MV and VI events as separate outcomes of interest.

As our primary analysis, we utilized logistic regression to determine the association, depicted as odds ratios (ORs), between patients experiencing their first CE within 48 h of being transferred to the PICU and in-hospital mortality. We adjusted for the following confounders: patient's age, gender, number of prior comorbidities (categories of 0, 1, and >1), and hospital site. Prior comorbidities were derived using the Pediatric Complex Chronic Condition classification system for diagnosis codes from all prior hospital admissions (24–26). Data did not contain any missing elements with regard to outcome, exposure, and confounders. As a secondary analysis, we determined the association between mortality and CE that occur in <12 h and 12–48 h of PICU transfer after adjusting for confounders. Patients who experienced a CE event in the <12-h time interval were not considered for the 12–48 h interval. We conducted two sensitivity analyses using these time windows: (a) by the origin of transfer (ward vs. ED) and (b) for different patient age groups (i.e., <2, 2–5, 6–11, 12–17 years). As a third level of analysis, we determined if the fully adjusted association between mortality and experiencing a CE varied by time by considering 12-h increments from the time of PICU transfer (<12, 12–24, 24–36, 36–48 h). As before, patients who experienced a CE in an earlier time period were not considered for the subsequent time periods. Finally, we conducted a sensitivity analysis by type of event (MV or VI) within each 12-h interval to detect any time-varying associations for each event type. A two-sided *p*-value of <0.05 was used to indicate statistical significance. All analyses were performed using R, version 3.6 (R Project for Statistical Computing, Vienna, Austria).

## 3. Results

### 3.1. Study population

Out of 10,741 transfers to the PICU from the ED or ward across both hospitals, 1,408 (13.1%) patients experienced a CE in the PICU. Patients who experienced a CE in the PICU after transfer were similar in age and sex to patients who did not experience a CE (see Table 1). PICU transfer patients who had a CE were more likely to be black (64% vs. 55%, *P* < 0.05), less likely to be Hispanic (17% vs. 22%, *P* < 0.05), more likely to have more than one prior comorbidity (34% vs. 18%, *P* < 0.05), were more likely to die in the hospital (5.8% vs. 0.2%, *P* < 0.05), and have a longer length of hospital stay (10 days vs. 3 days) than patients who did not experience a CE. Out of 1,408 patients with a CE, 1,260 (90%) patients were mechanically ventilated, and 427 (30%) were administered vasoactive drugs during their PICU stay.

**Table 1:** Comparison of characteristics and outcomes for pediatric intensive care unit (PICU) transfer patients who experienced a critical event from those who did not.

	PICU transfers who experience a critical event (*n* = 1,408)	PICU transfers who did not experience any critical events (*n* = 9,333)	*P*-value
Age, years (mean, sd)	5.9 (5.7)	5.9 (5.6)	0.766
Female, (*n*, %)	603 (42.8%)	4,039 (43.3%)	0.773
Race, (*n*, %)			
Black	900 (63.9%)	5,177 (55.5%)	<0.05
White	309 (21.9%)	2,425 (26.0%)	
Other	199 (14.1%)	1,731 (18.5%)	
Hispanic, (*n*, %)	238 (16.9%)	2,093 (22.4%)	<0.05
Number of prior comorbidities, (*n*, %)			
0	820 (58.2%)	6,857 (73.5%)	<0.05
1	108 (7.7%)	835 (8.9%)	
>1	480 (34.1%)	1,641 (17.6%)	
Died in-hospital, (*n*, %)	82 (5.8%)	18 (0.2%)	<0.05
Hospital length of stay, days, (median, IQR)	10 (5–20)	3 (2–5)	<0.05
Experienced invasive mechanical ventilation event during PICU stay, (*n*, %)	1,260 (89.5%)	–	
Administered vasoactive drugs during PICU stay, (*n*, %)	427 (30.3%)	–	

### 3.2. Analysis of CE occurrence

**Figure 1:**
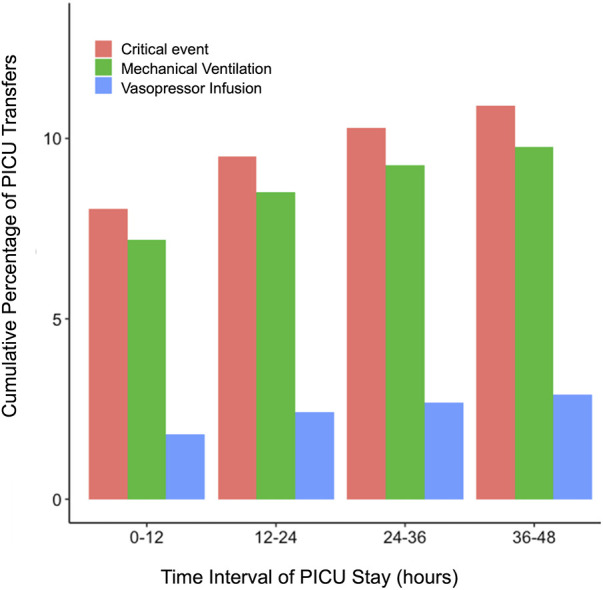
Cumulative graph depicting the occurrence of critical events, initiation of invasive mechanical ventilation, and vasoactive infusion at different time points after PICU transfer.

Figure 1 depicts the cumulative percentage of PICU transfers with their first CE against ordered time intervals from when the patient was transferred to the PICU. Approximately 8.1% of PICU transfers experienced an event within the first 12-h of PICU transfer, accounting for 62% of CEs, whereas approximately 5% of PICU transfers (or about 38% of all CEs) experienced events between 12 and 48 h of PICU transfer. Figure 1 also depicts cumulative event rates for MV and VI events. Approximately 7.2% of PICU transfers had an MV event within the first 12 h, and 4.5% of PICU transfers had MV events after the first 12 h. Approximately 1.8% of PICU transfers experienced VI events within the first 12 h, and 2.2% of PICU transfers experienced VI events after 12 h. Additionally, approximately 40% of MV and 55% of VI events among all PICU transfers occurred after the first 12 h.

### 3.3. Mortality and occurrence of CE

After adjusting for possible confounders, experiencing a CE within 48 h of being transferred to the PICU increased the risk of mortality [OR 12.40 (95% CI: 8.12–19.23, *P* < 0.05)] compared to patients who did not experience any CE within 48 h. Supplementary Table S1 depicts the OR (with 95% CI) for all variables in the model.

Patients who experienced their first CE within the first 12 h of being transferred to the PICU had a very high likelihood of mortality [OR 11.32 (95% CI: 7.51–17.15, *P* < 0.05)], in comparison to patients who did not experience a CE within the first 12 h of PICU transfer, after adjusting for possible confounders. However, experiencing an initial CE 12–48 h of PICU transfer also increased the patient's risk for in-hospital mortality [OR 2.84 (95% CI: 1.40–5.22, *P* < 0.05)]. Supplementary Table S2 depicts the association between mortality and experiencing critical events for the origin of PICU transfer, i.e., from ward vs. from ED. Patients transferred from the ED had a high risk of mortality if they experienced a CE within the first 12 h of mortality, but the risk remains moderately high for ED transfer patients who experience CE after the first 12 h (≤12 h: OR 25.62 [95% CI: 14.00–49.27, *P* < 0.05], 12–48 h: OR 2.69 [95% CI:0.80–6.82, *P* = 0.06]). Notably, patients who were transferred from the ward had a consistently high risk of mortality if they experienced a CE regardless of the time of the event (≤12 h: OR 4.34 [95% CI: 2.29–8.03, *P* < 0.05], 12–48 h: OR 2.36 [95% CI: 0.92–5.26, *P* < 0.05]). The results of our sensitivity analysis by age group are given in Supplementary Table S3. The association between in-hospital mortality and experiencing a CE within the first 12 h of PICU transfer was strongest among patients less than 2 years of age. Events experienced between 12 and 48 h after PICU transfer from ward or ED were consistently associated with mortality across all age groups, but with varying effect sizes on account of low frequency.

**Figure 2:**
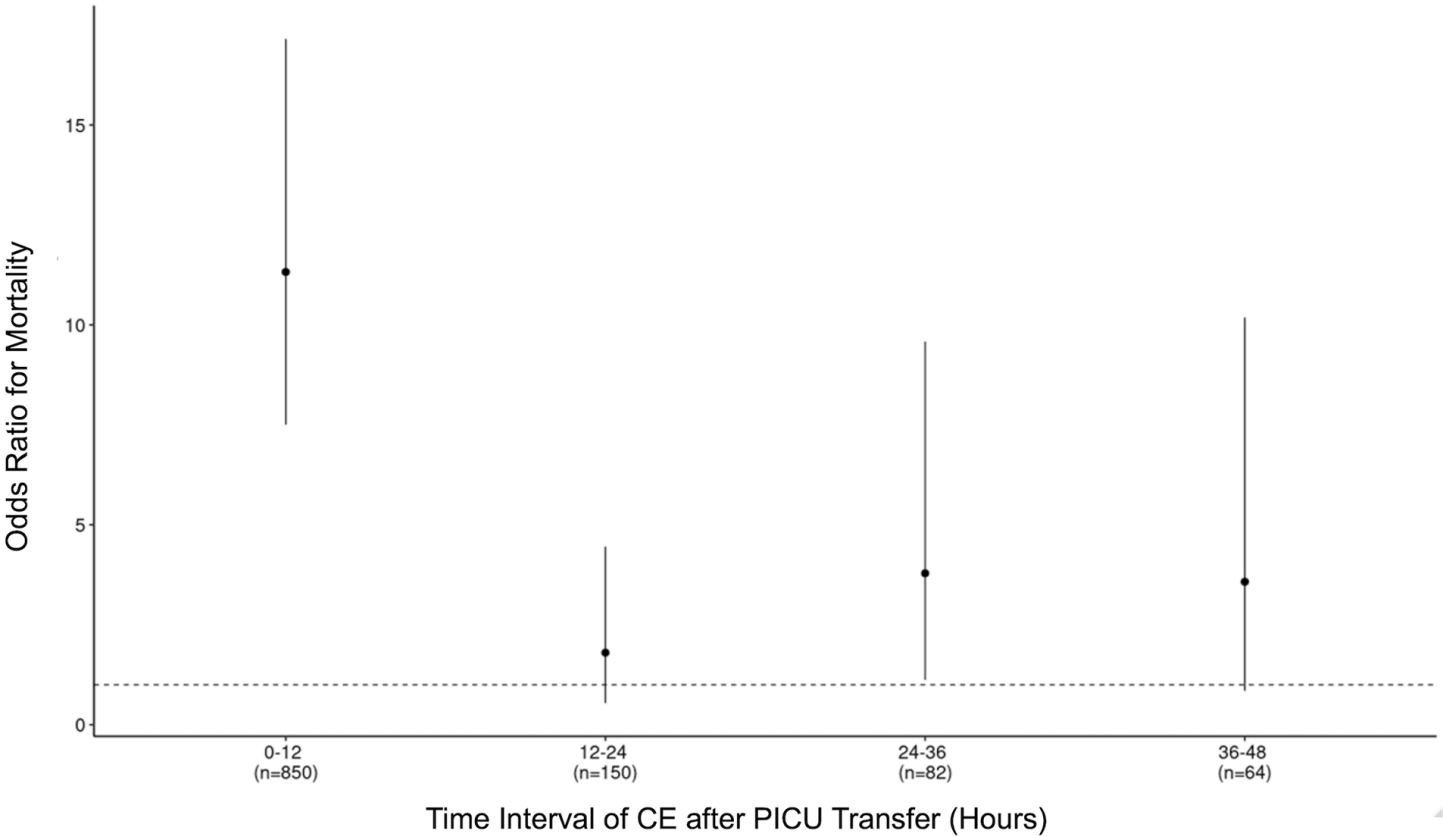
Adjusted odds ratio (OR) for mortality with 95% confidence interval (CI) for experiencing a critical event at different time points after PICU transfer.

Figure 2 depicts the ORs for mortality after experiencing an initial CE within incremental 12-h time intervals from 0 to 48 h after PICU transfer. As shown, CEs within the first 12 h were of highest incidence (*n* = 850) and highest OR. Patients who experienced CEs within 12-h intervals from 12 to 48 h after PICU transfer were at varying risks for mortality and statistical significance (12–24 h: OR 1.80 [95% CI: 0.54–4.45, *P* = 0.26], 24–36 h: OR 3.79 [95% CI: 1.12–9.58, *P* < 0.05], 36–48 h: OR 3.57 [95% CI: 0.85–10.18, *P* < 0.05]). Supplementary Figure S1 depicts the association between experiencing an initial MV or VI event and in-hospital mortality for different time intervals during a PICU stay. As can be seen, the risk of mortality after an MV event tracked closely with the risk of mortality after a CE in terms of distribution across periods (≤12 h: OR 10.32 [95% CI: 6.80–15.63, *P* < 0.05], 12–24 h: OR 2.08 [95% CI: 0.62–5.15, *P* = 0.16], 24–36 h: OR 4.19 [95% CI: 1.24–10.63, *P* < 0.05], 36–48 h: OR 4.92 [95% CI: 1.44–12.75, *P* < 0.05]). However, the association between experiencing a VI event and mortality was similar across ≤12 and 12–24 h [≤12 h: OR 20.27 [95% CI: 12.45–32.30, *P* < 0.05]; 12–24 h: OR 13.13 [95% CI: 5.97–26.43, *P* < 0.05]), but decreased for 24–48 h [24–36 h: OR 4.78 [95% CI:0.75–17.16, *P* < 0.05]; 36–48 h: OR 6.32 [95% CI: 0.98–23.23, *P* < 0.05]).

## 4. Discussion

In this retrospective multicenter study, we demonstrate that CEs, defined as the initiation of invasive mechanical ventilation or administration of vasoactive drugs, often occur within 48 h of being transferred to the PICU from the ward and the ED. After adjusting for confounders, patients who experience these events are at increased risk for mortality. Notably, the risk is highest for ED-to-PICU transferred patients who experience CEs within the first 12 h of PICU transfer. The increased mortality risk persisted for CEs between 12 and 48 h, although not uniformly when considering individual 12-h time intervals. Our study provides support for the use of CEs within 48 h of PICU transfer as an additional metric to evaluate the effectiveness of clinical interventions or quality improvement initiatives within the ward or the ED tertiary-care settings.

The “critical deterioration” metric, i.e., CEs or death within 12 h of a ward to PICU transfer, was created to evaluate the effectiveness of implementing pediatric rapid response systems (RRS) (6, 7). Since standard metrics used to evaluate adult RRSs, such as cardiac arrests or resuscitation events, are less common in children, studies focusing on the implementation of RRS or other interventions in a pediatric hospital have either extended to multicenter data or delayed evaluation for an extended time to acquire adequate power (27, 28). In contrast, CD events were observed to be more common (1.52 events per 1,000 non-ICU patient days) compared to cardiac or respiratory arrests (0.18 events per 1,000 non-ICU patient days). These events were associated with in-hospital mortality, as patients who experienced CD events were more likely to die than patients who did not in an unadjusted analysis (16.7% vs. 1.3%). The definition of CD includes an event component (patient must experience ventilation or be administered vasoactive drugs) and a time component (patient must experience these events in the first 12 h).

Over time, studies have expanded on this definition. For example, in a recent study, Hussain et al. defined a proximal outcome of “emergency transfer” as a transfer from an acute care floor to a PICU where the patient received intubation, inotropes, or ≥3 fluid boluses in the first h after arrival or before transfer and demonstrated through unadjusted analysis that these events were associated with poor patient outcomes (23). Follow-up studies compared emergency transfers with CD events and supported their use as focal points around which clinical decision support tools could be optimized (29, 30).

Our study expands on the prior body of work by investigating the association between mortality and CEs, defined as invasive mechanical ventilation and vasoactive infusion events, within 48 h of PICU transfer from the ward or ED and after adjusting for potential confounders. In our study population, we observed that most CEs occur within 12 h of PICU transfer and lead to a high risk for mortality, thereby supporting prior studies but adjusting for possible confounding. However, our study demonstrated that 38% of CEs in our cohort occurred between 12 and 48 h of PICU transfer and were also associated with an increased risk for mortality. In addition, we found that patients with the highest mortality risk were those with a transfer from the ED and experienced a CE within 12 h (indicative of a population arriving at the ED severely ill and requiring immediate critical care services). The association wanes for other ED patients who experience a CE between 12 and 48 h of PICU transfer, with point estimates still indicating an increased risk for mortality. Interestingly, the mortality risk was relatively uniform for patients transferred from the floor regardless of when they experienced their first CE. We further observed a strong positive association between mortality and CEs across all age groups when experiencing a CE in the first 12 h. The association was positive but not statistically significant for a 12–48 h CE across all age groups. Finally, analyzing the association between mortality and events (both CE and individual MV/VI events) across incremental 12-h intervals demonstrated that the risk was non-uniform regarding point estimates and statistical significance. We note that although the odds-ratio point estimates for all our models are greater than one, our sub-group analyses are likely to be underpowered due to low sample size and event rates. Taken together, our study provides initial evidence that, in addition to CD and emergency transfers, evaluation studies focusing on the impact of ward- and ED-based quality initiatives or clinical decision support tools could benefit from using the additional power offered by CEs within 48 h of PICU transfer. For example, using 48-h CE as a secondary outcome to evaluate the clinical impact of a new clinical decision support tool designed for early recognition of deterioration on the ward might lead to important findings that may otherwise be missed if the evaluation was limited to the primary outcomes of CD or emergency transfers.

Several groups have also developed and implemented early warning scores for identifying children at risk for deterioration to lower the risk of mortality and long-term morbidity in hospitalized children (16, 31, 32). However, most of these tools utilize a direct ward-to-PICU transfer as a definition of deterioration and are limited to the ward. A recent single-center study demonstrated that CEs occur throughout the hospital (ED: 19%, ward: 17%, ICU: 64%) (33). These observations, combined with the results from this study, suggest that using critical events as the primary outcome for early warning tools may lead to continuous risk assessment of a child throughout their entire hospital stay.

Our study has limitations. First, as a retrospective analysis, there may be inaccurate documentation regarding the timing of events. However, we used broad 12-h intervals to study the association between critical events and mortality to compensate for mislabeling. A second limitation is that residual confounding may remain unaccounted for in our analysis. For example, even though we adjust for prior comorbidities, there may be effects from conditions such as multiorgan dysfunction syndrome (MODS) that may occur before or within a critical event (34, 35). The casual interplay between MODS and CEs and their association with mortality remains to be investigated (36, 37). Third, we eliminated patients who experienced a CE in the ED or ward prior to transfer and focused only on the first CE event. Further, our MV/VI stratified analyses did not exclude prior occurrences of the other event type (VI/MV). Analyses to determine the increased risk of mortality from experiencing multiple CE events, either in the PICU or throughout the hospital stay, are areas of future work. Another limitation is that we only considered invasive mechanical ventilation and vasoactive administration in this study. Although these events closely align with the original definition of CD, the impact of expanding the definition of critical events to include other events, such as cardiopulmonary resuscitation, renal replacement therapy, etc., needs to be further studied.

Conversely, the association between critical events and other outcomes, such as the onset of a new disease, also needs to be further studied. Finally, although we limited our analysis to 48-h CEs, some may result from disease progressions during ICU stay. Therefore, studies comparing overall rates of 48-h CEs before and after the implementation of a ward or ED clinical quality improvement initiative are encouraged to use chart review to eliminate events that may not be impacted.

In conclusion, we demonstrate that the occurrence of a 48-h CE was associated with mortality in patients transferred to the PICU. Variation was observed in the association's magnitude and statistical significance across different intervals, patient sub-populations, and event types. Our study provides evidence for using 48-h CEs as a metric for evaluating the effectiveness of clinical quality improvement tools and for risk stratification and prediction.

## Data Availability

The data analyzed in this study is subject to the following licenses/restrictions: HIPAA restrictions, Data Use Agreements. Requests to access these datasets should be directed to moguss@medicine.wisc.edu.
