# Supplementary material for: Association between mortality and critical events within 48 hours of transfer to the pediatric intensive care unit
Source: Front Pediatr. 2023 Dec 21;11:1284672. doi: 10.3389/fped.2023.1284672 (PMC10768058; doi:10.3389/fped.2023.1284672)
Supplement: Supplementary file 1 [file Datasheet1.docx]

Supplementary Material

# Supplementary Figures and Tables

## Supplementary Tables

**Supplementary Table 1:** OR with 95% CI for all variables in the primary logistic regression model.

| **Variable** | **OR (95% CI)** | **P-value** |
| --- | --- | --- |
| Age | 1.06 (1.02-1.10) | <0.05 |
| Male | 1.02 (0.68-1.54) | 0.92 |
| 1 prior comorbidity | 3.04 (1.56-5.56) | <0.05 |
| >1 prior comorbidities | 2.77 (1.78-4.31) | <0.05 |
| Site = Loyola | 0.38 (0.20-0.66) | <0.05 |
| CE within 48 hours of PICU transfer | 12.40 (8.12-19.23) | <0.05 |

**Supplementary Table 2:** Adjusted odds ratio for mortality when experiencing a critical event after PICU transfer based on the origin of PICU transfer.

| **Origin of ICU transfer** | **Odds Ratio (95% CI)**  $\leq$**12 hours** | **Odds Ratio (95% CI)**  **12-48 hours** |
| --- | --- | --- |
| Emergency Department (n= 900) | 25.62 (14.00-49.27)* | 2.69 (0.80-6.82) |
| Ward (n= 508) | 4.34 (2.29-8.03)* | 2.36 (0.92-5.26)* |

*****P<0.05

**Supplementary Table 3:** Adjusted odds ratio for mortality when experiencing a critical event after PICU transfer based on the age of the patient.

| **Age Group** | **Odds Ratio (95% CI)**  **0-12 hours** | **Odds Ratio (95% CI)**  **12-48 hours** |
| --- | --- | --- |
| <2 years (n=485) | 33.81 (13.48-97.91)* | 2.49 (0.39-8.72) |
| 2-6 years (n=315) | 9.01 (3.07-26.65)* | 4.30 (0.64-17.07) |
| 6-12 years (n = 283) | 9.52 (4.17-22.04)* | 2.41 (0.54-7.58) |
| >12 years (n = 325) | 8.46 (4.04-17.33)* | 2.68 (0.76-7.32) |

*P<0.05

## Supplementary Figures

**Supplementary Figure 1.** Adjusted odds ratio for mortality when experiencing a mechanical ventilation or vasopressor infusion at different time points within 48 hours of PICU transfer
